# Clinical Severity and Hospitalization Burden of Influenza Subtypes: A Cross-Sectional Analysis From a Tertiary Care Center in North India

**DOI:** 10.7759/cureus.98302

**Published:** 2025-12-02

**Authors:** Shiv Prakash Sharma, Basu Kanwar Rathore, Manoj Meena, Neha Sharma

**Affiliations:** 1 Preventive Medicine, Rajasthan University of Health Sciences College of Medical Sciences (RUHS-CMS), Jaipur, IND; 2 Respiratory Medicine, Institute of Respiratory Diseases, Sawai Man Singh (SMS) Medical College, Jaipur, IND; 3 Microbiology, Bharatpur Medical College, Bharatpur, IND

**Keywords:** clinical symptoms, h1n1 subtype, h3n2 subtype, hospitalization, influenza a virus, influenza b virus

## Abstract

Introduction

Influenza viruses cause a significant annual burden of respiratory illness. While virological surveillance tracks circulating subtypes, data on the comparative clinical severity and healthcare burden imposed by different influenza subtypes in North India are limited. This study aimed to compare the clinical presentation and hospitalization rates associated with influenza A(H1N1)pdm09, A(H3N2), and B/Victoria lineages and to identify factors associated with hospitalization.

Methodology

A cross-sectional analysis was conducted on 598 laboratory-confirmed influenza cases identified through prospective surveillance of 7,231 patients with influenza-like illness (ILI) from July 2019 to June 2020. Subtyping was performed using real-time reverse transcription-polymerase chain reaction (rRT-PCR). Data on demographics, clinical symptoms, and hospitalization status were collected. Associations between subtypes and clinical outcomes were analyzed using the chi-square test, and factors associated with hospitalization were identified using binary logistic regression.

Results

Influenza A(H3N2) was the predominant subtype, accounting for 379 (63.4%) cases, followed by A(H1N1)pdm09 with 121 (20.2%) and influenza B/Victoria with 98 (16.4%). The overall hospitalization rate was 223 (37.3%). Hospitalization rates did not differ significantly between subtypes. The presence of shortness of breath was the strongest independent predictor of hospitalization (OR: 9.21, 95% CI: 6.23-13.62, p < 0.001). Patients under 5 years (OR: 2.15, 95% CI: 1.21-3.82, p = 0.009) and those over 59 years (OR: 1.82, 95% CI: 1.11-2.98, p = 0.018) also had significantly higher odds of hospitalization. A(H1N1)pdm09 infected a significantly higher proportion of individuals aged 41-59 years (31, 25.6%) compared to A(H3N2) (66, 17.4%; p = 0.037).

Conclusions

The presence of dyspnea and extremes of age were the primary drivers of hospitalization in this influenza cohort, not the infecting viral subtype. These findings underscore the critical importance of closely monitoring patients with respiratory distress and prioritizing vaccination for the very young and elderly. Hospital preparedness for seasonal influenza should focus on managing respiratory complications across all age groups, irrespective of the dominant circulating strain.

## Introduction

Influenza remains a formidable global public health challenge, responsible for significant annual morbidity and mortality and a strain on healthcare systems [1]. The dynamic nature of influenza A and B viruses, characterized by constant antigenic evolution, leads to the co-circulation of different subtypes and lineages each season, including A(H1N1)pdm09, A(H3N2), and the B/Victoria and B/Yamagata lineages [2]. While virological surveillance effectively tracks these circulating strains to inform vaccine composition, understanding the clinical implications of this diversity is crucial for frontline physicians and hospital administrators [3].

Evidence suggests that influenza subtypes can differ in their impact, influenced by factors such as intrinsic virulence, population immunity, and the age profile of affected individuals [4]. For instance, A(H3N2)-predominant seasons have often been associated with increased morbidity and mortality in elderly populations, while A(H1N1)pdm09 has been noted to cause severe disease in younger and middle-aged adults [5]. Similarly, the clinical burden of influenza B viruses, once considered milder, is now recognized as substantial, particularly in pediatric populations [6].

In India, with its diverse climate and complex influenza seasonality, regional data on subtype-specific disease severity are essential yet sparse [7]. Most published studies from the region focus on epidemiological trends and virological characterization, leaving a gap in the understanding of the direct clinical consequences of infection with different subtypes. This study aimed to bridge this gap by analyzing the clinical presentation and hospitalization burden associated with the predominant influenza subtypes and lineages circulating in a tertiary care setting in North India, and to identify key demographic and clinical factors predictive of hospital admission. The findings are intended to provide clinicians and public health officials with evidence to better anticipate patient needs and optimize resource allocation during influenza outbreaks.

## Materials and methods

Study design and population

This cross-sectional analysis was conducted as part of a larger laboratory-based surveillance study at a tertiary care hospital in Jaipur, Rajasthan, North India. The study period spanned 12 months, from July 2019 to June 2020. The analysis included all patients who presented with influenza-like illness (ILI), defined as an acute respiratory infection with a measured fever of ≥38°C and cough, with symptom onset within the previous 10 days, and who tested positive for an influenza virus. A total of 598 laboratory-confirmed influenza-positive cases were included in this clinical analysis.

Ethical considerations

The study protocol was approved by the Institutional Ethics Committee of S.M.S. Medical College and Attached Hospitals, Jaipur (Approval No. 365/MC/EC/2020). Written informed consent was obtained from all participants or their legal guardians prior to enrollment.

Data collection and laboratory methods

Demographic data (age, gender), clinical symptoms (fever, cough, sore throat, nasal catarrh, shortness of breath), and hospitalization status (inpatient/IPD or outpatient/OPD) were recorded at the time of sample collection. Throat swab samples were collected in viral transport media and tested for influenza A and B viruses using real-time reverse transcription-polymerase chain reaction (rRT-PCR) following established CDC/WHO protocols [8]. Influenza A-positive samples were further subtyped for A(H1N1)pdm09 and A(H3N2), while influenza B-positive samples were characterized for B/Victoria and B/Yamagata lineages using subtype-specific primers and probes.

Statistical analysis

Data were analyzed using IBM SPSS Statistics for Windows, Version 20.0 (Released 2011; IBM Corp., Armonk, NY, USA). Descriptive statistics were used to summarize demographic and clinical characteristics. Categorical variables were presented as frequencies and percentages (n, %). The associations between influenza subtypes and categorical variables (e.g., symptoms, hospitalization, age groups) were assessed using the chi-square test. To identify factors independently associated with hospitalization, a binary logistic regression analysis was performed, including age group, gender, presence of shortness of breath, and influenza subtype as covariates. A p-value of less than 0.05 was considered statistically significant.

## Results

Distribution of influenza subtypes and clinical presentation

During the study period, 598 patients with laboratory-confirmed influenza were included in the analysis. The majority of infections were caused by influenza A(H3N2) (379, 63.4%), followed by influenza A(H1N1)pdm09 (121, 20.2%) and influenza B/Victoria (98, 16.4%). The B/Yamagata lineage was not detected. The clinical presentation and hospitalization status of patients are detailed in Table 1. Fever and cough were universal symptoms present in all 598 (100%) patients. Other common symptoms included nasal catarrh (536, 89.6%) and sore throat (527, 88.1%). Shortness of breath was reported in 224 (37.5%) patients. A total of 223 (37.3%) patients required hospitalization. There were no statistically significant differences in the prevalence of specific clinical symptoms or in hospitalization rates across the three influenza subtypes.

**Table 1:** Clinical presentation and hospitalization status of patients by influenza subtype. p-values were calculated using the chi-square test for comparisons across the three influenza subtypes.

Characteristic		Overall (N = 598)	A(H3N2) (n = 379)	A(H1N1)pdm09 (n = 121)	B/Victoria (n = 98)	Test statistic	p-value
Symptoms, n (%)	Fever	598 (100)	379 (100)	121 (100)	98 (100)	-	-
	Cough	598 (100)	379 (100)	121 (100)	98 (100)	-	-
	Sore throat	527 (88.1)	344 (90.8)	98 (81.0)	85 (86.7)	χ² = 9.51	0.050
	Nasal catarrh	536 (89.6)	350 (92.3)	102 (84.3)	84 (85.7)	χ² = 8.78	0.067
	Shortness of breath	224 (37.5)	148 (39.1)	40 (33.1)	36 (36.7)	χ² = 1.62	0.510
Hospitalization, n (%)		223 (37.3)	148 (39.1)	40 (33.1)	35 (35.7)	χ² = 1.54	0.464

Age and gender distribution

The age and gender distribution of patients infected with different subtypes are shown in Table 2. The 20-40 age group was the most affected across all subtypes (233, 39.0%). A notable finding was that a significantly higher proportion of patients infected with A(H1N1)pdm09 belonged to the 41-59 age group (31, 25.6%) compared to those infected with A(H3N2) (66, 17.4%; p = 0.037). Regarding gender, a male predominance was observed across all subtypes, with 349 (58.4%) of the total cases being male, though this distribution did not differ significantly by subtype.

**Table 2:** Age and gender distribution of patients by influenza subtype. p-values were calculated using the chi-square test for comparisons across the three influenza subtypes.

Characteristic		Overall (N = 598)	A(H3N2) (n = 379)	A(H1N1)pdm09 (n = 121)	B/Victoria (n = 98)	Test statistic	p-value
Age group, n (%)	0-4 years	84 (14.0)	51 (13.5)	17 (14.0)	16 (16.3)	χ² = 15.2	0.230
	5-19 years	62 (10.4)	40 (10.6)	8 (6.6)	14 (14.3)		
	20-40 years	233 (39.0)	139 (36.7)	41 (33.9)	53 (54.1)		
	41-59 years	102 (17.1)	66 (17.4)	31 (25.6)	5 (5.1)		
	≥60 years	117 (19.6)	83 (21.9)	24 (19.8)	10 (10.2)		
Gender, n (%)	Female	249 (41.6)	161 (42.5)	50 (41.3)	38 (38.8)	χ² = 0.45	0.800
	Male	349 (58.4)	218 (57.5)	71 (58.7)	60 (61.2)		

The results of the binary logistic regression analysis to identify factors associated with hospitalization are presented in Table 3. The presence of shortness of breath was the strongest independent predictor, with patients reporting this symptom having over nine times higher odds of being hospitalized (OR: 9.21, 95% CI: 6.23-13.62, p < 0.001). Age was also a significant factor; compared to the 20-40 years reference group, children under 5 years had more than double the odds of hospitalization (OR: 2.15, 95% CI: 1.21-3.82, p = 0.009), and adults aged 60 years and above had 1.82 times higher odds (OR: 1.82, 95% CI: 1.11-2.98, p = 0.018). Influenza subtype and gender were not significantly associated with hospitalization in the multivariate model.

**Table 3:** Factors associated with hospitalization among influenza-positive patients (binary logistic regression). This table presents the results of a binary logistic regression analysis with hospitalization as the dependent variable. The model included all variables listed in the table. The Hosmer-Lemeshow test indicated a good model fit (p = 0.415).

Factor	Category	Adjusted odds ratio (aOR)	95% confidence interval (CI)	p-value
Age group	20-40 years	Reference	-	-
	0-4 years	2.15	1.21 - 3.82	0.009
	5-19 years	1.42	0.76 - 2.66	0.274
	41-59 years	1.29	0.79 - 2.11	0.309
	≥60 years	1.82	1.11 - 2.98	0.018
Gender	Female	Reference	-	-
	Male	1.07	0.77 - 1.49	0.688
Shortness of breath	No	Reference	-	-
	Yes	9.21	6.23 - 13.62	<0.001
Influenza subtype	A(H3N2)	Reference	-	-
	A(H1N1)pdm09	0.79	0.51 - 1.23	0.297
	B/Victoria	0.90	0.55 - 1.46	0.662

## Discussion

This hospital-based study provides a detailed clinical perspective on the burden of different influenza subtypes in North India. The principal finding is a substantial hospitalization rate of 37.3% across all influenza subtypes, underscoring the significant burden seasonal influenza places on tertiary healthcare resources. This rate is considerable and highlights influenza as a cause of serious illness requiring inpatient care, consistent with studies from other regions that have documented substantial hospitalization burdens associated with both influenza A and B viruses [9,10].

The most critical finding from our analysis is that the infecting influenza subtype was not an independent predictor of hospitalization. Instead, the clinical factor of shortness of breath and the demographic factors of very young and old age were the primary drivers of hospital admission. The presence of dyspnea was an exceptionally strong predictor, increasing the odds of hospitalization more than ninefold. This aligns with clinical reasoning, as respiratory compromise is a key indicator of severe influenza and a common reason for inpatient management [11,12]. This finding provides an evidence-based triage criterion for physicians in outpatient settings; a patient with confirmed influenza and shortness of breath should be evaluated for admission with high priority.

The significantly elevated odds of hospitalization at the extremes of age reinforce well-established global patterns of influenza severity [1,5]. Our data confirm that in this North Indian cohort, children under five and older adults remain the most vulnerable populations for severe outcomes, likely due to less robust immune responses and a higher prevalence of underlying risk factors. This reinforces the public health imperative to increase vaccine coverage in these groups.

A key demographic observation was the significant predilection of the A(H1N1)pdm09 virus for adults aged 41-59 years. This pattern has been observed since the 2009 pandemic and is often attributed to the concept of "original antigenic sin," where the first influenza infection in childhood imprints a lifelong immune response, potentially leaving certain middle-aged cohorts with less protective immunity against specific viral proteins in the A(H1N1)pdm09 strain [13,14]. While this group did not have significantly higher odds of hospitalization in our multivariate model, their high rate of infection is of clinical and public health importance, as it identifies a specific, economically productive age group that may be disproportionately affected during A(H1N1)pdm09-predominant seasons [15].

The study has several limitations. Its design as a single-center study may limit the generalizability of the findings to other regions or community settings. The lack of data on comorbidities, vaccination status, and detailed vital signs prevented a more nuanced analysis of risk factors for severe disease. Furthermore, the study period coincided with the emergence of COVID-19, and the public health measures implemented may have influenced healthcare-seeking behavior and influenza transmission in the latter part of the study.

## Conclusions

This analysis demonstrates that the decision to hospitalize a patient with influenza in this North Indian cohort was driven primarily by the presence of respiratory distress and patient age, not by the specific infecting viral subtype. The consistently high hospitalization rate across all subtypes calls for sustained hospital preparedness for seasonal influenza surges. For clinicians, these findings emphasize that shortness of breath is the most critical clinical sign warranting hospital evaluation. For public health officials, the data reinforce the need to protect the very young and elderly through vaccination. Future research incorporating data on comorbidities and oxygen saturation will further refine our understanding of severity risk factors.

## References

[1] Iuliano AD, Roguski KM, Chang HH (2018). Estimates of global seasonal influenza-associated respiratory mortality: a modelling study. Lancet.

[2] Petrova VN, Russell CA (2018). The evolution of seasonal influenza viruses. Nat Rev Microbiol.

[3] Fitzner J, Qasmieh S, Mounts AW (2018). Revision of clinical case definitions: influenza-like illness and severe acute respiratory infection. Bull World Health Organ.

[4] Tokars JI, Olsen SJ, Reed C (2018). Seasonal incidence of symptomatic influenza in the United States. Clin Infect Dis.

[5] Simonsen L, Clarke MJ, Schonberger LB, Arden NH, Cox NJ, Fukuda K (1998). Pandemic versus epidemic influenza mortality: a pattern of changing age distribution. J Infect Dis.

[6] Paul Glezen W, Schmier JK, Kuehn CM, Ryan KJ, Oxford J (2013). The burden of influenza B: a structured literature review. Am J Public Health.

[7] Chadha MS, Potdar VA, Saha S (2015). Dynamics of influenza seasonality at sub-regional levels in India and implications for vaccination timing. PLoS One.

[8] (2017). World Health Organization. WHO information for the molecular detection of influenza viruses. https://www.who.int/teams/global-influenza-programme/laboratory-network/quality-assurance/eqa-project/information-for-molecular-diagnosis-of-influenza-virus.

[9] Sellers SA, Hagan RS, Hayden FG, Fischer WA 2nd (2017). The hidden burden of influenza: a review of the extra-pulmonary complications of influenza infection. Influenza Other Respir Viruses.

[10] Wong J, Layton D, Wheatley AK, Kent SJ (2019). Improving immunological insights into the ferret model of human viral infectious disease. Influenza Other Respir Viruses.

[11] Garg S, Jain S, Dawood FS (2015). Pneumonia among adults hospitalized with laboratory-confirmed seasonal influenza virus infection-United States, 2005-2008. BMC Infect Dis.

[12] Aleem MA, DeBord KR, Ahmed M (2024). Incidence of hospitalization due to influenza-associated severe acute respiratory infection during 2010-2019 in Bangladesh. Influenza Other Respir Viruses.

[13] Gostic KM, Ambrose M, Worobey M, Lloyd-Smith JO (2016). Potent protection against H5N1 and H7N9 influenza via childhood hemagglutinin imprinting. Science.

[14] Bai Y, Guo Y, Gu L (2023). Additional risk factors improve mortality prediction for patients hospitalized with influenza pneumonia: a retrospective, single-center case-control study. BMC Pulm Med.

[15] Miron VD, Săndulescu O, Streinu-Cercel A (2024). Age, comorbidity burden and late presentation are significant predictors of hospitalization length and acute respiratory failure in patients with influenza. Sci Rep.

